# Educational values and challenges of i-STEM project-based learning: A mixed-methods study with data-transformation design

**DOI:** 10.3389/fpsyg.2022.976724

**Published:** 2022-11-22

**Authors:** Chi-Cheng Chang, Yi-Kai Chen

**Affiliations:** Department of Technology Application & Human Resource Development, National Taiwan Normal University, Taipei, Taiwan

**Keywords:** educational value, data-transformation design, mixed-methods research, project-based learning, robot

## Abstract

Integrated science, technology, engineering, and mathematics (STEM) embedding project-based learning (i-STEM PjBL) is still faced with challenges, and its educational values have not been revealed, which is what the study aimed to explore. Participants consisted of 48 freshmen from a senior high school, including 27 male students and 21 female students. The open-ended questionnaire and the interview for the students were administrated after the i-STEM PjBL. The qualitative data were converted into quantitative data counted by the occurrence frequencies of the codes. The results based on the integration and comparison of the open-ended questionnaire and interview outcomes showed that i-STEM PjBL provided students with positive educational values (including learning acquisition, performance, and perception), but there were also learning challenges in the process. Learning acquisition focused on the basic structure and components of a robot, principles of robot motion, hull structure, principles of sailboat navigation, and skills of designing and assembling sailboats. Learning performance referred that students were satisfied with their hands-on performances and confident of their abilities to perform better in similar disciplines, but did not learn well on programming. Learning perception indicated that students felt interested in i-STEM PjBL materials could acquire knowledge and skills from various fields, PjBL could be helpful to complete works, and principles could be helpful in practice, while programming design learning materials were not enough. Learning challenges indicated that students were unfamiliar with the usage of tools and hands-on operation, and they also felt challenged by programming. Students' feedback can be taken as references to modify and improve i-STEM PjBL and the materials in the future.

## Introduction

### STEM educational values and challenges

Science, technology, engineering, and mathematics (STEM) education have been an important trend throughout the world. If learning STEM separately, it can cause students' isolated knowledge system and influence their learning outcomes. STEM is closely related and should be integrated and studied together (Kelley et al., 2020; Awad, 2021). One of the important educational values in STEM is interdisciplinary integration; in other words, students can be assisted to tackle real-world challenges by making meaningful connections and integrating knowledge across disciplines (Johnson, 2012).

Previous studies indicate that STEM education can improve students' performance of cognition, affect, skills, and so on (Johnson, 2012; Kelley et al., 2020), which is recognized as another educational value. However, many STEM learning materials only include one or two disciplines, lacking connection, and integration among disciplines; besides, how to integrate STEM remains vague (Awad, 2021). Although Kelley et al. (2020) raised a conceptual framework of integrated STEM (i-STEM), it lacks clear integration approaches. Cheng et al. (2020) classified disciplines in STEM learning activities but did not explain how different disciplines were linked or integrated.

Educational values refer to the purposes or roles of education for people and society and extend various definitions. The output value of education refers to the value of educational activities or products that can meet social, political, economic, and cultural demands; the process value of education refers to the value of meeting certain educational aims or processes (Young, 2000). Educational values can be explored individually (such as developing personal potential, promoting self-realization, self-transcendence, etc.), socially, politically, and economically or include the five aspects: cognitive, health, social, personal, and national (Ayasrah et al., 2020); this study focuses on educational values from an individual perspective centered on cognition.

Investigating educational values and challenges in learning materials and learning is helpful to understand whether STEM can practice effectively and whether it needed any adjustments (Smith and Ragan, 2005). In sum, research on STEM learning and materials can be helpful to highlight STEM educational values and identify challenges.

### Theoretical basis of i-STEM learning and educational values and challenges

As situated cognition theory designates, students can acquire knowledge and skills from real situations and learn how to apply them in reality (Brown et al., 1989). Based on situated cognition theory, i-STEM learning attempts to improve students' science, computational thinking, and problem-solving abilities through scientific inquiry and engineering design which integrated multiple disciplines (Kelley et al., 2020). The engineering design process can be used as a context for problem-solving in reality, allowing students to practice situated cognition and situational learning, and further acquire knowledge and skills (Kelley et al., 2020). Another i-STEM learning concept is based on mathematics, enabling students to learn scientific concepts and apply technology through the interpretation of engineering problems (Yakman, 2008). Galadima et al. (2019) divided i-STEM learning into single discipline, combine disciplines, multiple disciplines, engineering projects, and fully i-STEM disciplines. One kind of integration is accomplished by project-based learning (PjBL). In sum, educational values of i-STEM learning can be explored from integration, cognition, meta-cognition, skill, attitude, affection, ability, and behavior.

There are some challenges in i-STEM learning and materials. Even though technology and engineering were added in recent years, it still lacks complete integration among the four disciplines (Kelley et al., 2020). Some i-STEM learning only contains one or two disciplines without clear connection or integration in between, and they lack descriptions of integrated methods or detailed lesson plans (Kelley et al., 2020; Awad, 2021). Although some i-STEM learning situations include different disciplines, the learning purposes or content only focus on a single discipline (Kelley et al., 2020; Mejias et al., 2021). Some i-STEM learning content is difficult to guarantee that they can cover all disciplines (Kelley et al., 2020); for example, some scientific and mathematical theories are difficult to provide a real engineering design or a technology application environment, which reduces the practicality of i-STEM education. The crosscutting connection among various disciplines is challenging, it is hard for teachers to teach fluently; furthermore, it is also challenging for students to apply knowledge from different disciplines to solve problems. Consequently, some crosscutting connection is implicit rather than performed apparently, which means it has already been included in the discipline. It is difficult for students to connect concepts across fields if they do not have sufficient knowledge in a single discipline; similarly, students are unable to apply knowledge naturally from a single subject to the context of knowledge integration (Kelley et al., 2020). Teachers who have neither adequate multidisciplinary knowledge nor multidisciplinary teaching skills can be incapable of teaching i-STEM (Nadelson et al., 2012).

### Theoretical basis of STEM PjBL and educational values and challenges

As constructivism emphasizes, learners can construct new or meaningful knowledge proactively through prior knowledge and real-world experience (Elliott et al., 2000). Based on constructivism, STEM materials integrating PjBL attempt to integrate various disciplines for learners to solve problems and construct experiences in real world (Bicer et al., 2019). The integration concept of STEM learning and PjBL is highly compatible. Integrating or embedding PjBL into STEM learning and materials, known as i-STEM PjBL and materials, can make use of the content of multiple disciplines and combine the PjBL strategy to promote its educational value. STEM materials integrated with PjBL (or i-STEM PjBL) can enhance students' affective mathematics engagement, including mathematical self-acknowledgment and value (Lee et al., 2019), engineering design capabilities (Yen and Chang, 2020), motivation, self-efficacy, learning STEM interests, and perceive the fact that learning STEM is important to their career development (Kuo et al., 2019). Moreover, i-STEM PjBL improves the positive attitude on divergent thinking and self-cognition to solve problems creatively (Bicer et al., 2019); i-STEM PjBL makes students proud of their works and teachers satisfied with how STEM content is integrated to develop the work (Alves et al., 2019). Interdisciplinary PjBL provides students with experiential learning and practical abilities (Murray et al., 2020). PjBL can motivate students to actively seek solutions through project tasks, which improves STEM interdisciplinary PjBL. Through PjBL, students apply tools and technology to explore scientific phenomena and solve engineering problems, which is an effective strategy for STEM learning materials (Yen and Chang, 2020). To sum up, the educational values of STEM PjBL can be split into cognition, meta-cognition, skills, attitudes, affection, abilities, behaviors, etc., to investigate.

PjBL solves problems or constructs works step by step through scientific methods, including asking and redefining questions, searching for relevant information, planning and designing, constructing relevant equipment, collecting data, analyzing data, concluding, looking for solutions, sharing research findings, etc. (Krajcik et al., 2003). PjBL emphasizes authentic activities, including planning, studying, practicing, experimenting, and so on; through the process of discovering and solving problems, products are finally built and evaluated (Kanter, 2010; Mohr-Schroeder et al., 2014). PjBL enables students to explore relevant scientific and mathematical concepts autonomously; through cooperation and communication, which is emphasized by PjBL, students' learning can be well-enhanced (Venville et al., 2000). Hands-on PjBL can enhance problem-solving abilities and physics (Hong et al., 2012) and significantly improve middle school students' motivations for STEM, making them more willing to engage in learning (Mohr-Schroeder et al., 2014; Awad, 2021).

PjBL provides students with a learning environment to fully communicate with each other, such as cooperating with peers, arguing for concepts, challenging peers' ideas, sharing concepts, and so on (Krajcik et al., 2003). In PjBL activities, students have to cooperate with peers in order to grasp the best performance and enhance educational values; the cooperation is involved with peers, teachers, and other learning environments, which are helpful in understanding (Schneider et al., 2002). The output of the work is an important accomplishment of PjBL (Fernandes et al., 2012). PjBL stresses that participants obtain the relevant knowledge, skills, and abilities to solve problems through the process of constructing works (Krajcik et al., 2003; Hong et al., 2012). Educational values (e.g., learning acquisition, learning performance, and learning perception) of i-STEM PjBL and materials are not revealed (Alves et al., 2019; Kuo et al., 2019; Lee et al., 2019). Moreover, i-STEM PjBL and materials are still faced with challenges.

### Research objectives and questions of the study

To sum up, this study aimed to examine the educational values and challenges of i-STEM PjBL. The research questions are as follows: (1) What are the educational values (including learning acquisition, performance, and perception) of i-STEM PjBL that the students perceive? (2) What are the challenges of i-STEM PjBL that the students perceive? The importance of this study lies in understanding i-STEM PjBL and materials, and its educational values and challenges. The research results can be taken as references for STEM educators

## Methodology

### Participants

Participants consisted of 48 freshmen from a senior high school, including 27 male students and 21 female students with an average age of 15. Although these students had a basic understanding of mathematics and physics, the majority of them lacked practical experience. Students were assigned into 16 groups at random, and there were 3 students in each group.

### Methods

This study adopted the approach of mixed-methods research with a data-transformation design (Creswell and Plano Clark, 2011). This approach entails analyzing data, which are collected through one research method and then convert into another form of data to analyze again. For example, after collecting qualitative data, they would be analyzed in qualitative and quantitative approaches; eventually, comparing and integrating the two results (Teddlie and Tashakkori, 2009). In order to allow the original qualitative data to be reused, this study converted the qualitative data into quantitative data counted by the occurrence frequencies of the codes or themes. Consequently, the study can compare and integrate the differences between the two analysis results to obtain more effective information to explain the problem, and thus increase the depth of this investigation (Patton, 1990; Creswell and Plano Clark, 2011). The results of the two forms of data analysis can be cross-checked and supplemented to eliminate data inadequacies or omissions. The results of the two investigations can also be used to support each other, yielding more comprehensive, diversified, and distinct interpretations.

### Data collection and analysis

The open-ended questionnaire would be filled by students after the i-STEM PjBL, and items were referred to the questionnaire by Fernandes et al. (2012), including educational values (learning acquisition, learning performance, and learning perception) and learning challenges. Learning acquisition referred to what knowledge and skills students had gained, learning performance referred to how students performed in specific knowledge and abilities, and learning perception referred to students' psychological reactions to the i-STEM PjBL and materials. Learning challenges were difficulties and frustrations students faced in the learning process. The interview took place after the open-ended questionnaire was completed, and each group selected one person at random for the 30-min interview. The interview questions were based on the outcomes of the open-ended questionnaire that students filled out, in order to get more in-depth answers from students.

Interview comments were initially transcribed to texts by the researcher/analyst, later submitted to the co-analyst to review, and finally submitted to the interviewee to check before finalizing the draft; this was the triangulation test of data accuracy (Denzin and Lincoln, 1994). The researcher/analyst and the co-analyst analyzed the open-ended questionnaire outcomes and the interview texts first, and then they submitted them to the instructor or students to review, and this method was recognized as the triangulation test of the data analysis or the researcher/analyst (Denzin and Lincoln, 1994).

This study adopted MAXQDA for coding and quantifying, including searching keywords, counting numbers, categorizing, clustering, comparing, and so on. First, four questions in the open-ended questionnaire—learning acquisition, learning performance, learning perception, and learning challenges (the first three items belonged to educational values) (Fernandes et al., 2012)—were taken as main codes; inducing and extracting terms with similar meaning of each main code as sub-codes and counting numbers of main codes and sub-codes (detailed in the Results Section). Among the four main codes, the total frequency and student number of similar terms which occurred in learning acquisition were the highest. This demonstrated that learners were convinced of the learning acquisition of i-STEM learning and materials.

This study adopted MAXQDA to calculate the interrater reliability (IR) of codes by percent agreement. The overall IR between the researcher/analyst and the co-analyst was 0.75, which was improved by the instructor's review and discussion until they completely agreed with each other. The formula of IR of codes is shown as follows: IR = M / (N1 + N2) (McHugh, 2012), where IR refers to interrater reliability calculated by percent agreement, M refers to the number of two analysts' mutual agreement, and N1, N2 refers to the number of each analyst's agreement. Besides, SPSS was applied to statistics (detailed in the Results Section).

### i-STEM PjBL materials

#### Learning materials and objectives

The i-STEM PjBL materials and learning objectives (1) were based on a practical concept, “self-made sailboat,” and to enhance participants' learning motivations; (2) adopted low-cost self-made kits in the materials; and (3) embedded with PjBL to improve the integration of the materials. The learning objectives were to learn how to design and construct a sailboat robot that could move on the surface of water and follow the route.

#### Material kits and component kits

**Figure 1:**
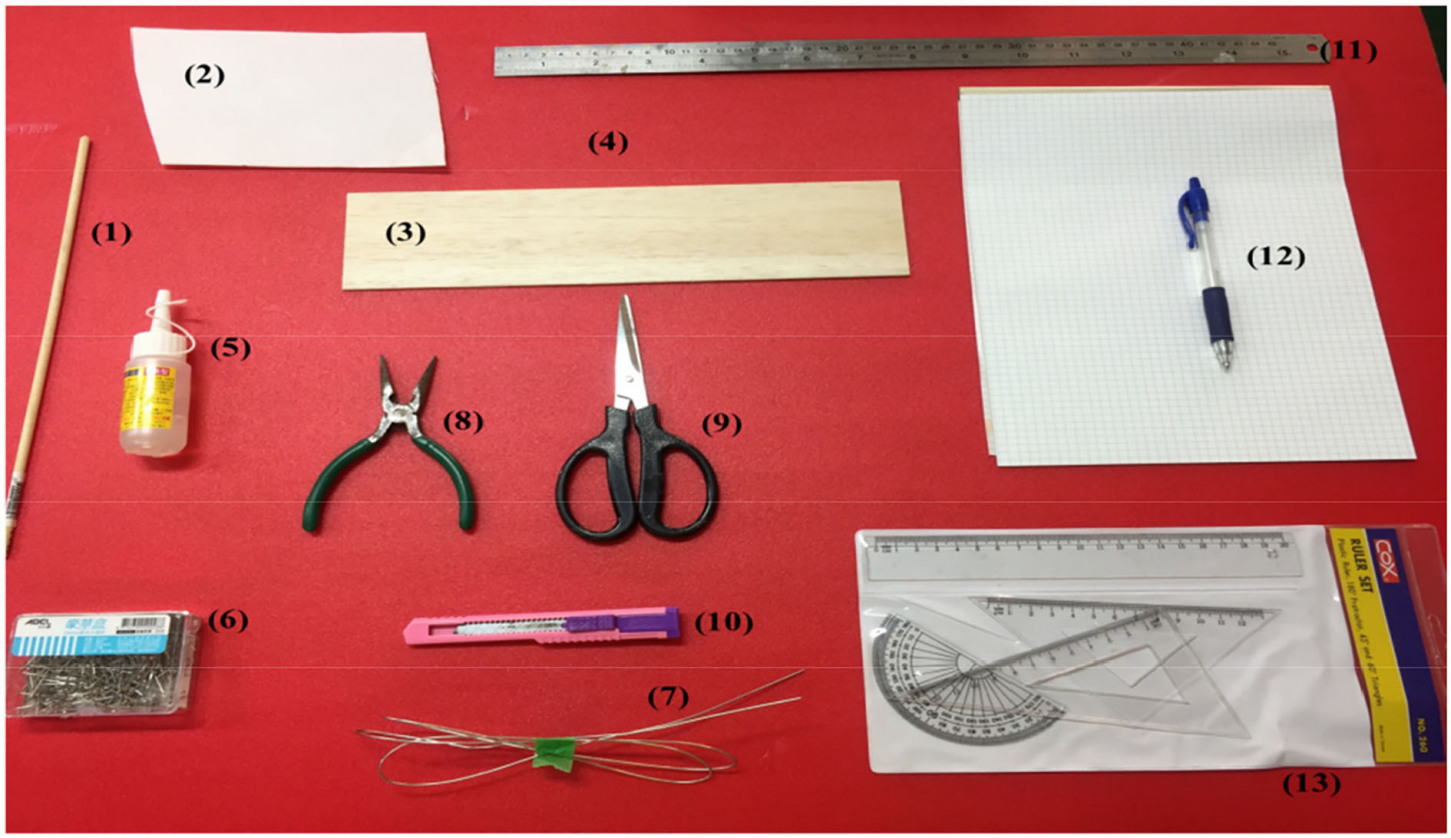
Materials and tools for making the hull of a sailboat.

Figure 1 includes materials and tools for making the hull of a sailboat. Materials were bars (1) for making the mast of a sailboat, thin (2) and thick (4) foam board, and cardboard (3). These materials were stuck and fixed by Styrofoam glue (5), pin (6), and thin wire (7). Pliers (8), scissors (9), and blades (10) cut and modified the above materials. Ruled paper (12) and rulers (11, 13) measured the size of materials. These materials and tools were inexpensive and easily available, also corresponding with the spirit of maker movement.

**Figure 2:**
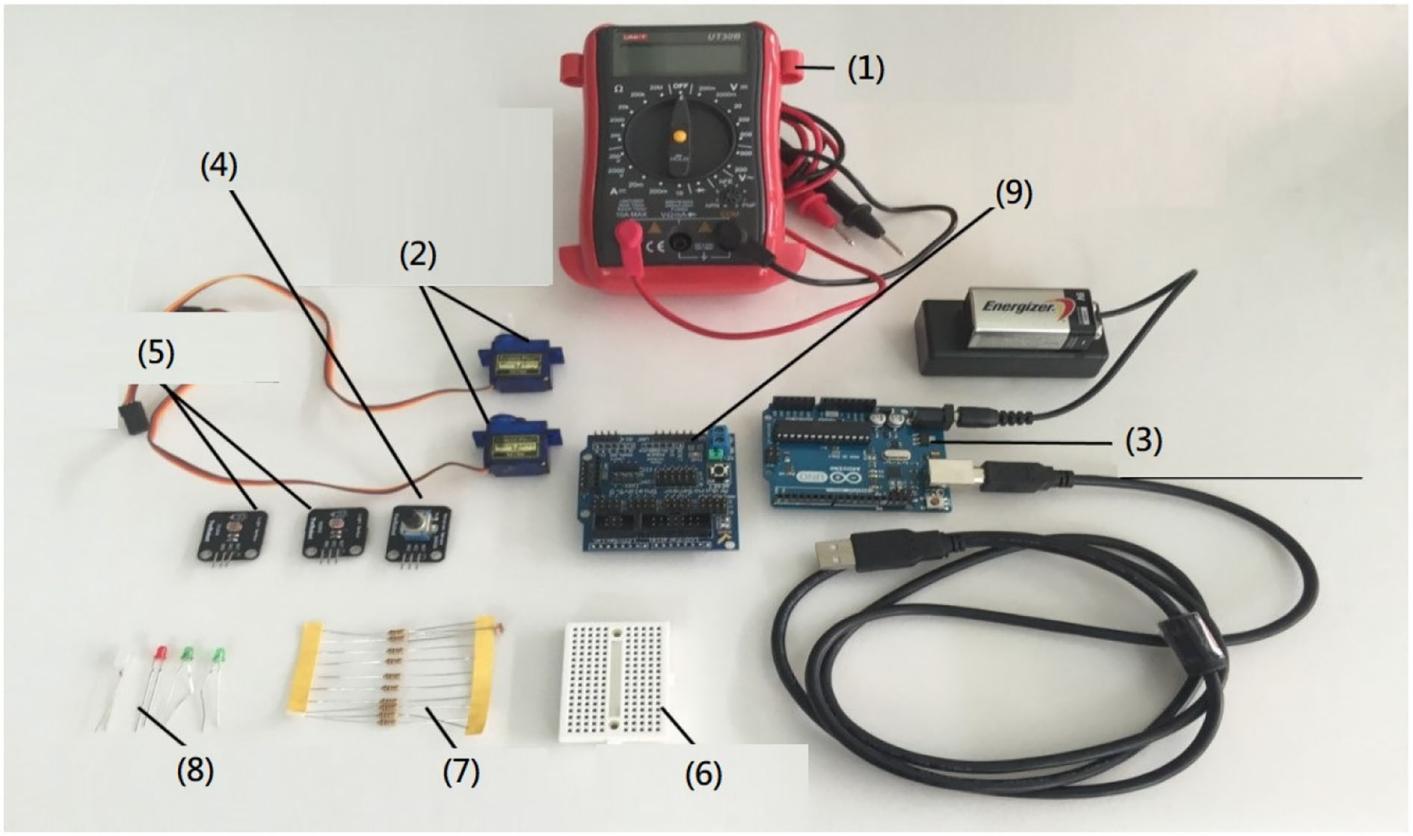
Components and measuring tools.

Figure 2 includes components and measuring tools. A multimeter (1) measured the circuit; a breadboard (6) linked all components together, such as resistors (7), light emitting diode (LED) (8), and so on, and checked whether the circuit worked.

The reasons why components used Arduino circuit board (3) included as follows: Arduino circuit board was easy to use and learn for beginners; Arduino circuit board was highly compatible with other components, for example, sensors and various components could be linked; besides, expansion board (9) provided Arduino circuit board, such as servomotors (2), with extra function. Variable resistor (4) had two fixed pins and one moving pin, and the resistance value would be changed through moving between the moving pin and two fixed pins. Adjusting the variable resistor to change the speed of the servomotor could produce a potentiometer circuit with different ratios. Photo resistor (5) was a resistor to measure the illumination; by sensing the light, students could use a flashlight to drive a sailboat robot on the surface of the water.

Compared to other robot kits on the market, the above kits were less expensive and easier to operate. Such materials were convenient to be supplemented and substituted, so as to decrease students' loss due to missing components and materials.

#### Material framework and learning

According to constructivism and Galadima et al. (2019), this study was based on the idea “integrate and link various disciplines by the project” (detailed in the Literature Review Section), and practiced PjBL. In this PjBL, the emphasis on design and practice enabled students to construct the final products of sailboat robots by applying previous knowledge and related technical and engineering abilities. These hands-on activities facilitated the integration of knowledge and skills, and the integration of interdisciplinary concepts (Nathan et al., 2013).

STEM knowledge framework in the learning materials is shown in Table 1. Science included the basic unit “hull structure,” “principles of sailboat navigation,” “basic structure and components of robot,” and “principles of robot motion.” Technology included “the usage of tools (such as multimeter)” and “information technology application (using pads or computers)”; furthermore, in the advanced stage “controlling microcontroller unit” and “controlling server” were included. Engineering was based on engineering design skills, allowing students to experience the process of “problem definition,” “data collection,” “concept formation,” “modeling,” “feasibility analysis,” “evaluation,” “review,” “decision,” “communication and sharing,” and so on (Atman et al., 2007). Mathematics included the basic level of estimating and calculating “numbers and quantities,” “statistics and probabilities,” and “geometry.”

**Table 1:** STEM knowledge framework and PjBL activities.

**Activity and time**	**PjBL stage**	**STEM knowledge and skill**			
		**Science (S)**	**Technology (T)**	**Engineering (E)**	**Mathematics (M)**
The 1st week 1.1. Introduce sail angle and push force	1. Exploration 2. Practice 3. Reflective evaluation	1. Hull structure 2. Principles of sailboat navigation	1. Sailboat materials and the usage of tools and devices 2. Apply information technology (draft a sailboat route map and components of force diagram)	–	1. Numbers and quantities 2. Statistics and probabilities
1.2. Introduce buoyancy, center of gravity and control	1. Exploration 2. Design 3. Practive 4. Reflective evaluation	1. Principles of sailboat navigation	1. Sailboat materials and the usage of tools and devices 2. Hull design, drafting, construction and test	–	1. Numbers and quantities 2. Geometry
The 2nd week 2.1. Introduce and test electronic components	1. Exploration 2. Practice	1. Robot structure	1. The usage of electronic components and tools		–
2.2. Introduce automatic control and programming	1. Exploration 2. Reflective evaluation	1. Principles of robot motion	1. Arduino circuit board and programming 2. Manipulate servo and linkage 3. Use variable resistors to control servomotor (programming)	–	–
The 3rd week 3.1. Design a programmable sailboat robot	1. Exploration 2. Design 3. Practive 4. Reflective evaluation	–	1. Control microcontroller 2. Control servomotor 3. Programming design 4. Assemble the hull of a sailboat robot (Arduino chip and hull)	1. Problematize sailboat navigation 2. Sailboat data collection 3. Form the concept of sailing	1. Numbers and quantities 2. Geometry
3.2. Construct a programmable sailboat robot	1. Exploration 2. Design 3. Practive 4. Reflective evaluation	–	1. Make sail, mast and servo linkage; assemble them to hull 2. Test the motion and programming among servomotor, sail and rudder	1. Modeling a sailboat 2. Feasibility analysis of a sailboat	–
The 4th week 4.1. Test and adjust programmable sailboat robot	1. Exploration	–	1. Test and adjust programming	1. Sailboat navigation evaluation 2. Sailboat navigation decision	–
4.2. Group competition	1. Reflective evaluation 2. Work-sharing	–	–	1. Communicate and share	–

**Figure 3:**
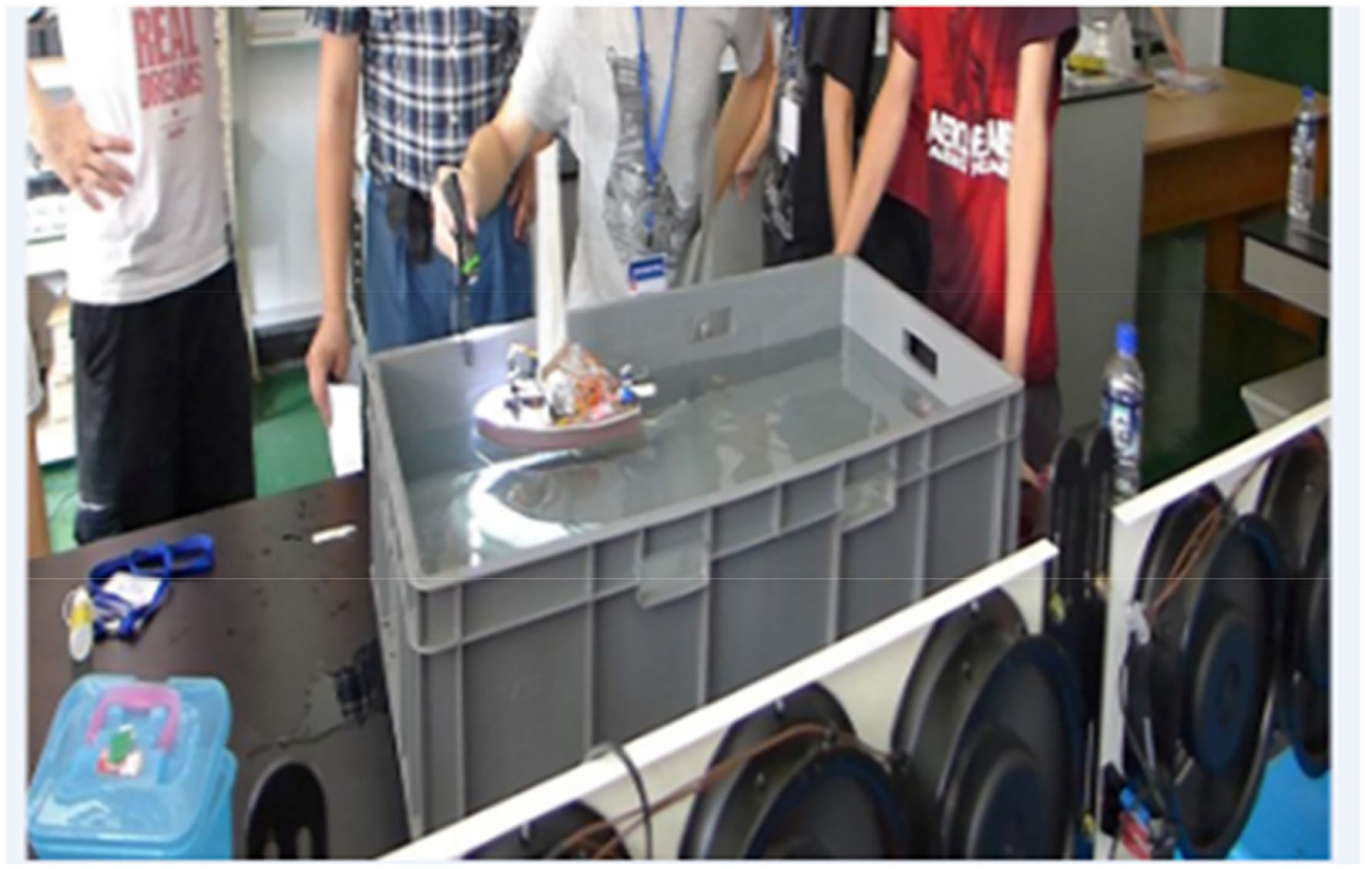
Sharing works—group competition.

Table 1 displays the PjBL activities and stages in each week. This study incorporated various types of PjBL, centering on design and practice. The stages included investigation (defining the problem and gathering information), design, practice (building equipment and collecting data, analyzing data, drawing conclusions, and identifying solutions), reflective evaluation, and work sharing (Figure 3) (Krajcik et al., 2003; Kanter, 2010; Mohr-Schroeder et al., 2014). To achieve horizontal interdisciplinary integration, knowledge from various disciplines was embedded in the associated PjBL activities. For example, in the 1st week, when students learned the invariant relationship between the sail angle and push force, it could be explained by Mechanics and Kinematics on this phenomenon and also supported by drafting and information technology on the invariant relationship. As shown in Table 1, in addition to horizontal interdisciplinary integration, there was also an emphasis on vertical interdisciplinary integration. That is, students had opportunities to constantly review and apply what they had learned. Taking science as an example, the knowledge system of sailboats and robots established by students in basic activities must be reviewed and reflected during PjBL activities, in order to form the knowledge basis of PjBL activities, which is consistent with constructivism.

## Results

### Quantifying qualitative data

The number of the main codes and sub-codes in students' open-ended questionnaires and interviews are listed in Table 2. Among the four main codes, the total frequency and student number of terms related to learning acquisition were the most, which revealed that students perceived that i-STEM learning and the materials brought them the greatest learning acquisition. The total frequency and student number of terms related to learning challenges were the least, which demonstrated that, in comparison with other dimensions, students perceived that i-STEM learning and the materials brought them fewer learning challenges.

**Table 2:** The number of times each coding appeared and test of goodness-of-fit (*N* = 40).

**Main codes**	**Sub-codes**	**Frequency**	**Total frequency**	**Student number**
Learning acquisition	Basic structure and components of robot	28	122	36
	Principles of robot motion	23		
	Hull structure	23		
	Principles of sailboat navigation	18		
	Skills of designing and assembling sailboat	30		
		$\chi_{\left(4\right)}^{2}=$3.66		
Learning performance	Students were satisfied with their hands-on performances	30	62	34
	Students were confident of their abilities to perform better in similar disciplines	22		
	Students did not learn well on programming	10		
		$\chi_{\left(2\right)}^{2}$ = 9.81^*^		
Learning perception	Students felt interested in i-STEM PjBL materials	32	92	36
	Students could learn interdisciplinary knowledge and skills	28		
	PjBL was helpful to complete works	22		
	Principles were helpful in practice	10		
		$\chi_{\left(3\right)}^{2}$ = 12.00^*^		
Learning challenges	Students were unfamiliar with the usage of tools	19	37	22
	Students were unfamiliar with hands-on operation	8		
	Students felt challenged by programming	10		
		$\chi_{\left(2\right)}^{2}$ = 5.57		
			$\chi_{\left(3\right)}^{2}$ = 51.99^**^	$\chi_{\left(3\right)}^{2}$ = 4.25

* p < 0.05,

** p < 0.01.

As shown in Table 2, the result of the test of goodness-of-fit indicated, the total frequency among the four main codes was significantly different [$\chi_{\left(3\right)}^{2}$ = 51.99, *p* < 0.01], but the student number of writing the terms related to the main or sub-codes did not reach significantly [$\chi_{\left(3\right)}^{2}$ = 4.25, *p* < 0.05]. This confirmed that the total frequency dispersed over the four aspects (main codes), while the student number was distributed adequately. The frequency among different sub-codes in learning perception and learning performance was significantly different [$\chi_{\left(3\right)}^{2}$ = 12.00, $\chi_{\left(2\right)}^{2}$ = 9.81, *p* < 0.05], but the frequency among different sub-codes in learning acquisition and learning challenges (main codes) was not significantly different [$\chi_{\left(4\right)}^{2}$ = 3.66, $\chi_{\left(2\right)}^{2}$ = 5.57, *p* > *0.0*5]. This confirmed that the frequency of sub-codes dispersed in learning perception and learning performance (main codes), while the frequency of sub-codes was distributed adequately in learning acquisition and learning challenges (main codes).

### Qualitative data analysis

With regard to learning acquisition, 90% (36/40) of students mentioned in their open-ended questionnaires or interviews, they acquired the basic structure and components of a robot, principles of robot motion, hull structure, principles of sailboat navigation, and skills of designing and assembling sailboats in i-STEM PjBL (as shown in Table 2). This acquisition corresponded to the concepts discussed in the learning materials. The result argued that i-STEM PjBL and the learning materials assisted students' learning and reinforced meaningful comprehension (Kanter, 2010).

“*I generally understood the concept of robot and principles of robot motion taught by the teacher, and later learned how to put them into practice*.” (Open-ended questionnaire)

“*I had little knowledge with hull structure and principles of sailboat navigation before, but I finally understood them in PjBL*.” (Interview)

In terms of learning performance, 85% (34/40) of students stated that they were satisfied with their hands-on performances and confident of their abilities to perform better in similar disciplines, but did not learn well on programming (as shown in Table 2). The reasons why they did not learn well were possibly caused by the insufficient content in the learning materials and the content was somewhat challenging for beginners; both of which were also raised in learning challenges.

“*I used to like operate by hand, and I understood the principle. So I assembled well in the process*.” (Open-ended questionnaire)

“*I didn't learn well on programming, and if I have a chance I will learn it better in the future*.” (Interview)

As for learning perception, 90% (36/40) of students expressed their learning perception or feeling. Learning perception included that students felt interested in i-STEM PjBL materials, they could acquire knowledge and skills from various fields, PjBL was helpful to complete works, and principles were helpful in practice (as shown in Table 2). Through hands-on manipulation, students literally “practiced” what they had learned from the teacher, and this condition was consistent with some research that emphasized the importance of hands-on manipulation and learning in reality (Galadima et al., 2019) and description in situated cognition theory.

“This lesson was arranged well, I could construct works with classmates, and also learned something from different aspects.” (Open-ended questionnaire)

“The principles learned in the beginning are full of useful information, and they are also applied in the later works, which is a very practical lesson.” (Interview)

With respect to learning challenges, 55% (22/40) of students indicated they faced learning challenges, including being unfamiliar with hands-on manipulation and the usage of tools (such as a multimeter) and being challenged by programming (as shown in Table 2). This suggested that students were still uneasy with learning by doing, on which PjBL emphasized; besides, more specific teaching mechanisms for the theory and usage of tools were still needed. This result was different from the fact that “hands-on PjBL was able to improve students' motivations” by Mohr-Schroeder et al. (2014) and Awad (2021), and it was possibly because some students had fewer hands-on experiences in junior high school. It was worth noting that during the interview, some students expressed their dissatisfaction with the learning content of programming. Due to the limited learning period of the i-STEM PjBL material, the programming on robots had been slightly simplified to reduce students' learning load. Students could mainly experience programming through teachers' presentations. In the future, when similar learning activities are undertaken and time permits, students would have chances to design robot programming codes.

“*I seldom operated the multimeter in school before, so I was unfamiliar with it. After the teacher's demonstration, I'm able to measure the voltage*.” (Open-ended questionnaire)

“*I had never studied programming before, and I think it is a little bit difficult, and the time and content are insufficient*.” (Interview)

## Discussion and conclusion

Although a large proportion of students were satisfied with their hands-on performance in the i-STEM PjBL, some students were unfamiliar with hands-on manipulation and had little knowledge of tool operation, signifying that hands-on manipulation possibly caused different perceptions for students from various backgrounds. Students claimed that the content of the programming on robots was insufficient, and they felt challenged and did not learn well, and this condition revealed that programming was an interesting yet complicated challenge for the freshman in senior high school.

The elements in i-STEM PjBL included the concept and the process of engineering design, but students did not mention “engineering design” directly, which was possibly because teachers did not emphasize the subject purposely. As students responded in learning perception, “they could gain interdisciplinary knowledge and skills,” “PjBL was helpful to complete works,” and “principles were helpful in practice,” these advantages could be achieved in the process of engineering design; the above advantages were corresponding with the idea “engineering design has been recognized as an effective way to integrate various fields in STEM” (Kelley et al., 2020; Yen and Chang, 2020). The aforementioned results also revealed that senior high school students were still unfamiliar with engineering design, which remained in the pre-engineering stage. The discussion can be taken as a reference to modify the i-STEM PjBL and materials.

The i-STEM PjBL provided students with positive educational values (including learning acquisition, perception, and performance), but there were also learning challenges in the process. Learning acquisition focused on the basic structure and components of robots, principles of robot motion, hull structure, principles of sailboat navigation, and skills of designing and assembling sailboats; learning performance referred that students were satisfied with their hands-on performances, confident of their abilities to perform better in similar disciplines, and they did not learn well on programming; learning perception included that students felt interested in i-STEM PjBL materials, they could acquire interdisciplinary knowledge and skills, PjBL was helpful to complete works, and principles were helpful in practice. “They can gain interdisciplinary knowledge and skills” is consistent with some research in the past; that is, robot learning involves various fields, which assists students in interdisciplinary integration (Benitti, 2012; Chatzopoulos et al., 2020). “Principles are helpful in practice” indicates that knowledge can be applied in practice, which is corresponding with “the process of robot construction allows students to apply knowledge” (Mistikoglu and Ozyalcin, 2010; Rihtaršič et al., 2015). Learning challenges referred that students were unfamiliar with the usage of tools and hands-on manipulation and felt challenged by programming.

This study purported to explore the educational values and challenges of i-STEM PjBL. The research contribution lies in comprehending and reflecting on educational values and challenges through a mixed-methods study with a data-transformation design, and the results can be utilized as references for STEM educators. i-STEM PjBL, in this study, was based on self-made sailboat robots using low-cost DIY kits and embedded PjBL to improve the idea of integration in the learning materials. According to constructivism, this study integrated STEM disciplines with PjBL. The framework of the i-STEM PjBL materials incorporated four dimensions, integrated interdisciplinary knowledge horizontally and vertically, and PjBL activities. These ideas can be taken as references for i-STEM learning materials design.

In terms of educational implications, in order to overcome these challenges, teachers should not only promote the significance of hands-on practices but also give students more hands-on opportunities. Apart from increasing the learning content and time for programming, teachers should provide additional programming examples and explanations, as well as strengthen tutorials. Although i-STEM PjBL contained the concepts and processes of engineering design, students did not mention “engineering design” in the open-ended questionnaire and the interview. Therefore, teachers should put more emphasis on the importance and concepts of engineering design.

Since this is a qualitative study with a limited number and feature of research samples, the research findings should not be overgeneralized. Future research can also solicit faculty opinions in order to broaden the scope of the studies. In the future, it is suggested to investigate the issues related to programming and engineering design in high school. Through researchers' reflection on the i-STEM PjBL and materials, learning challenges can be reduced, and the i-STEM PjBL and learning materials can be further modified and improved in the future.

## Data availability statement

The datasets presented in this article are not readily available because the data is potentially identifiable. Requests to access the datasets should be directed to C-CC, samchang@ntnu.edu.tw.

## Ethics statement

Ethical review and approval was not required for the study on human participants in accordance with the local legislation and institutional requirements. Written informed consent from the patients/ participants or patients/participants legal guardian/next of kin was not required to participate in this study in accordance with the national legislation and the institutional requirements.

## Author contributions

C-CC made substantial contributions to conceptualization, formal analysis, methodology, project administration, resources, supervision, validation, writing—original draft and critical revision, and writing—review and editing. Y-KC made substantial contributions to conceptualization, data acquisition and curation, data analysis and interpretation, investigation, methodology, validation, visualization, and writing—original draft. Both authors contributed to the article and approved the submitted version.

## Conflict of interest

The authors declare that the research was conducted in the absence of any commercial or financial relationships that could be construed as a potential conflict of interest.

## Publisher's note

All claims expressed in this article are solely those of the authors and do not necessarily represent those of their affiliated organizations, or those of the publisher, the editors and the reviewers. Any product that may be evaluated in this article, or claim that may be made by its manufacturer, is not guaranteed or endorsed by the publisher.
